# A new perspective on ancient Mitis group streptococcal genetics

**DOI:** 10.1099/mgen.0.000753

**Published:** 2022-02-28

**Authors:** Sophie Belman, Chrispin Chaguza, Narender Kumar, Stephanie Lo, Stephen D. Bentley

**Affiliations:** ^1^​ Parasites & Microbes, Wellcome Sanger Institute, Hinxton, UK; ^2^​ Department of Genetics, University of Cambridge, Cambridge, UK; ^3^​ Yale School of Medicine, New Haven, CT, USA

**Keywords:** ancient, genomics, *Streptococcus*, *pneumoniae*, Syltholm, metagenome, Mitis

## Abstract

Mitis group *Streptococcus* are human obligate bacteria residing in the nasopharynx and oral cavity. They comprise both commensal and pathogenic species with the most well-known being *Streptococcus pneumoniae –* a leading cause of meningitis and pneumonia. A primary difference between the commensal and pathogenic species is the presence of the polysaccharide capsule – a major virulence factor in *S. pneumoniae *, also present in other commensal species. Our current understanding of the evolutionary divergence of the pathogenic and commensal species has been inferred from extant strains. Ancient genomes can further elucidate streptococcal evolutionary history. We extracted streptococcal genome reads from a 5700-year-old ancient metagenome and worked towards characterizing them. Due to excessive within- and between-species recombination common among streptococci we were unable to parse individual species. Further, the composite reads of the ancient metagenome do not fit within the diversity of any specific extant species. Using a capsular gene database and AT-content analysis we determined that this ancient metagenome is missing polysaccharide synthesis genes integral to streptococcal capsule formation. The presence of multiple zinc metalloproteases suggests that adaptation to host IgA1 had begun and the presence of other virulence factors further implies development of close host–microbe interactions, though the absence of a capsule suggests an inability to cause invasive disease. The presence of specific virulence factors such as pneumolysin implies stable maintenance of such genes through streptococcal evolution that may strengthen their value as anti-pneumococcal vaccine antigens, while maintaining awareness of their potential presence in commensal species. Following from Jensen *et al.*’s initial analysis we provide historical context for this long time human nasopharyngeal resident, the Mitis group *Streptococcus *.

## Data Summary

We include all reference genomes in Table S9.

**Impact Statement**

We confirm the presence of the genus *Streptococcus* amongst an ancient metagenome generated by Jensen *et al.* [22]. We were unable to place the reads among the extant diversity of any specific species, implying the probable mixture of Mitis group streptococcal species. The capsular polysaccharide locus may have been imported into the Mitis group more recently than 5700 years ago, the presence of multiple zinc metalloproteases suggests that adaptation to host IgA1 had begun and the presence of other potential virulence factors further implies development of close host–microbe interactions, though the absence of a capsule suggests an inability to cause invasive disease. Understanding historical gene presence among Mitis group streptococci can improve our efforts to mitigate disease caused by these highly recombinogenic dynamic bacterial pathogens.

## Introduction

Ancient DNA (aDNA) provides an opportunity to better understand the ever-evolving relationship between humans and human-pathogens. The advancement of high-throughput sequencing, alongside methods to extract low-quality and low-quantity DNA has resulted in an increase of ancient pathogen genome studies [1–3]. Some prominent aDNA studies have provided insights into the emergence of *Yersinia pestis *, the global dissemination of *Mycobacterium tuberculosis *, and pig domestication and genomic exchange resulting in emergence of *Salmonella enterica* serovar Paratyphi C [4–7]. Many inferences we make regarding pathogen evolution are based on the crowns of the phylogeny, while inferring the roots. However, it is becoming clear that aDNA provides an opportunity to more accurately elucidate features such as the historical relationships between species, potential spillover events, virulence acquisition and geographical spread, among many other things.

The *Streptococcus* Mitis group of Viridans streptococci are bacteria which typically inhabit the human nasopharynx. The Mitis group includes approximately 20 species, some of which rarely cause disease (commensals) such as: *Streptococcus pseudopneumoniae *, *Streptococcus mitis *, *Streptococcus oralis* subsp. *oralis* and *Streptococcus infantis *, and the more commonly pathogenic *Streptococcus pneumoniae* (the pneumococcus) [8–10]. These species are highly similar both by classical microbiological methods and by genome sequence similarity – this is due in part to their promiscuous recombination both within and between species [11, 12].

The pneumococcus is commonly pathogenic and is a human-obligate opportunistic pathogen carried in the nasopharynx of 20–90 % of the population, varying by host age and geographical location [13–15]. Pneumococcal infections are usually asymptomatic but also serve as the prerequisite for a range of diseases including otitis media, meningitis, pneumonia and septicaemia [16]. The capsular polysaccharide locus is an important virulence factor in invasive pneumococcal disease and prevents phagocytosis [17–19]. Many pneumococcal genes are shared by other commensal species in the Mitis group, including capsular genes [20, 21]. Understanding the historical exchange of genes between these species can inform the potential for future recombination events and further shows how one species of this diverse group has become such a notable pathogen.

It is proposed that a common ancestor of the commensals of the Mitis group and the pneumococcus existed as long as 6–7 million years ago in the nasopharynx of a hominoid species [20]. The primary theory involves development of human IgA1, the primary driver of mucosal adaptive immunity. It is postulated that this drove divergence of the common ancestor of extant *Streptococcus* along separate pathogenic and a commensal paths; the former diversified and increased its genome plasticity while the latter stabilized its genome and lost genes associated with immune evasion [11, 12, 20]. This theory has been developed using genetic data of extant streptococcal strains. The recent identification of streptococcal genomic reads in an approximately 5700-year-old metagenome in Denmark provides an opportunity to further investigate the evolutionary history of extant *Streptococcus* and the characteristics of ancient strains [22].

Our aims here were to determine whether, as stated in Jensen *et al.* [22], a species which sits within the extant diversity of *S. pneumoniae* was present in a 5700-year-old metagenome. If not, what relatives of the Mitis group were present and which genes did these comprise? What virulence factors, if any, did these representatives of the ancient streptococcal species include? Furthermore, we specifically interrogated the presence of a capsular locus (*cps*) due to the relevance its evolutionary history may have to the current pneumococcal vaccine.

## Methods

### Metagenomic processing

**Fig. 1.**
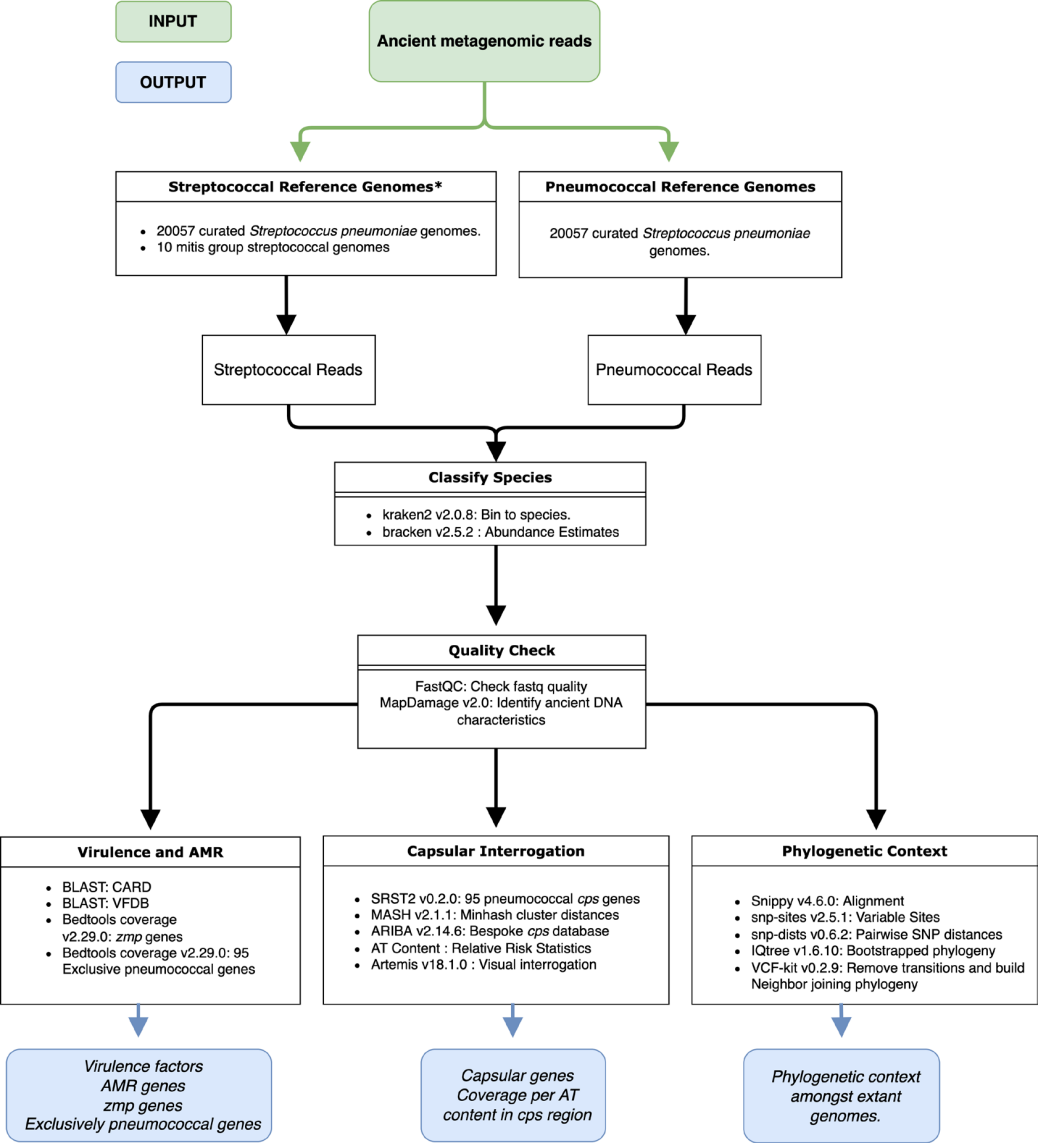
Schematic of the pipeline used for analysis of a 5700-year-old ancient streptococcal genome. We concatenated the reference genomes and aligned the ancient metagenomic reads (green) to them, extracting only those reads which aligned. We performed the rest of the analysis in parallel, resulting in the outputs (blue). *The Mitis group streptococcal genomes included the species *mitis*, *oralis*, *psuedopneumoniae* and a novel *mitis* ancestor with accession number OXCQ0000000.

Metagenome data were generated by Jensen *et al.* at the University of Copenhagen from an ancient birch pitch sample discovered near Syltholm on the island of Lolland in Denmark [22]. Jensen *et al.* performed initial analysis of streptococcal reads which led us to pursue this genome for further contextualization among extant strains [22]. We received the metagenome data after those reads aligning to the human genome version 37 (hg19) [10] were removed. We generated a content report using kraken2 v2.0.8 and bracken v2.5.2 [11, 12]. This identified several remaining human reads which we removed manually using the *Homo sapiens* taxonomy identification code (9606). in total, 1.17 % of the remaining metagenomic reads belonged to the family *Streptococcaceae *. Among the top 60 % of classifications, 4.34 % of the reads were binned to *S. mitis,* 3.54 % were binned to *S. pneumoniae* and 1.61 % were binned to *S. oralis *. Other top hits included species of the genera *Neisseria *, *Haemophilus* and *Delftia* (Table S1 available in the online version of this article). The entire pipeline used for downstream analysis is outlined in Fig. 1. Due to our particular interest in *S. pneumoniae* as the primary streptococcal causal agent for disease, we first attempted to extract only pneumococcal reads. The high rates of recombination both within and between species led us to repeat our analysis and extract all reads belonging to the streptococcal Mitis group. Both of these read extraction methods resulted in the majority of reads belonging to *Streptococcaceae* (Table S2).

To extract the genome reads of interest we aligned the metagenomic sample to two separate databases using BWA v0.7.17 and samtools v1.9 [13, 14]. One database, referred to as the pneumococcal database, contained 20047 concatenated, pneumococcus-only genomes from a global collection [23]. Due to the high rates of recombination both within the pneumococcal species and between sister species of the Mitis group we built another database to extract streptococcal reads which we refer to as the streptococcal database. This second database included the previous 20047 pneumococcal genomes as well as an additional three *S. mitis *, three *S. oralis* and three *S. pseudopneumoniae *. We also included a novel, pathogenic, Mitis group species in the streptococcal database described by Kirkeleite *et al*. [24]. Throughout the paper we refer to this isolate (with accession number OXCQ00000000) as *Streptococcus* OXCQ for clarity [17].

Genome reads extracted from both databases were run through mapDamage v2.0 to confirm the presence of key characteristics of aDNA including 5′ C to T and 3′ G to A substitutions and the accumulation of these over increasing read length [25].

We calculated heterozygosity and the heterozygous SNP to homozygous SNP ratio including only sites with mapping quality >30 and depth of coverage >10 [13].

The reads extracted from both databases were *de novo* assembled using megahit [26].

Statistics and plotting were performed in R v3.6.0.

### Phylogenetic context among extant strains

We built a phylogeny to determine if the genomic reads extracted using only the pneumococcal database fit within the extant pneumococcal diversity and could elucidate which extant pneumococcal strains might be ancestral. We used the reads extracted with the pneumococcal database and generated a multiple sequence alignment against reference *S. pneumoniae* PMEN1 (or ATCC700669), a Spanish serotype 23F reference genome (Accession: NC_011900) using snippy v4.6.0 [16]. The extant pneumococcal reads included a representative genome from each known global pneumococcal sequence cluster (GPSC) [27]. We used a *S. mitis* B6 outgroup. We generated a SNP alignment and SNP distance matrix using snp-sites v2.5.1 [28] and snp-dists v0.6.2 (https://github.com/tseemann/snp-dists).

We repeated the same phylogenetic pipeline to contextualize the Mitis group reads extracted from the streptococcal database by generating a multiple sequence alignment against the same PMEN1 reference genome also using snippy v4.6.0 [16]. This alignment included *Streptococcus* Mitis group species with *S. pneumoniae, S. mitis, S. oralis, S. infantis* and several *S. pseudopneumoniae* representatives. The extant streptococcal phylogeny was rooted to an *S. infantis* outgroup.

We removed all transitions due to their overrepresentation in aDNA as a result of DNA damage. We then built neighbour-joining trees for both ancient metagenomes extracted from the pneumococcal database and the streptococcal database using vcf-kit v0.2.9 [25, 29]. We also built phylogenetic trees using IQ-TREE v1.6.10 with 1000 bootstraps and a GTR model [18].

After determining the presence of heterozygosity in both metagenomes, we evaluated what the effect on its position within the extant diversity would be if it was a mix of multiple extant and ancient species. We pseudo-mixed genomic reads from extant *S. pneumoniae* and *S. mitis* and treated them as a single genome. We repeated the phylogenetic methods outlined above to build a phylogenetic tree and identify where this pseudo-mixed genome fitted in the extant streptococcal phylogeny.

All subsequent analysis was performed using the reads extracted using the streptococcal database.

All trees were visualized using FigTree v1.4.4.

### Pathogenicity and antimicrobial resistance

To identify the presence of genes associated with virulence and antimicrobial resistance genes we ran blast against the virulence factor database (VFDB) and the comprehensive antibiotic resistance database (CARD). We filtered the bitscore, coverage and identity with low cutoffs due to the low read depth of the ancient metagenome (bitscore >200, coverage >20 % and identity >80 %), confirming any putative genes by manually investigating the alignments. Furthermore, we assessed mapping coverage against genes designated as being specific to extant pneumococcal species (not present in commensal relatives; as described using 60 pneumococcal strains in Kilian *et al*. [30]) and thus more likely to contribute to pathogenicity. We calculated the fraction of sites with non-zero coverage using bedtools v2.29 with a minimum coverage of 20 % as the threshold for gene presence [19]. We additionally investigated the mapping coverage to zinc metalloproteases (*zmpA*/*iga*, *zmpB*, *zmpC*, and *zmpD*) due to their hypothesized contribution to the divergence and diversification of species [20]. Using the generated genome coverage data, we assessed coverage breadth for *zmp* genes from PMEN1 (Accession: NC_011900) and TIGR4 (Accession: NC_003028.3).

### Capsular locus

To determine whether there were genes for capsular polysaccharide biosynthesis (*cps*) within this metagenome, we utilized SRST2 v0.2.0 [31], MASH v2.1.1 [32] and ARIBA v2.14.6 [33]. The capsular genes found in the pneumococcus are also found in the genomes of Mitis group species [21, 34]. We first determined MASH kmer distances against 95 known pneumococcal capsular types. Furthermore, we used SRST2 to identify any short-read matches against concatenated FASTA files of 95 different pneumococcal serotypes [34]. To determine read coverage against individual capsular genes, we created an ARIBA database containing 54 capsular genes and five controls (Table S3). The capsular genes included 23 from PMEN1, 20 from TIGR4 and six from a GPSC81 Swiss non-typable genome (GCF_000817005.1). We additionally included *pspK*, and *aliB*-like genes (*aliC* and *aliD*), typically found in non-encapsulated *S. pneumoniae* (NESp) as well as in capsular regions of Mitis group species [21, 35]. The positive controls included four contigs from the *de novo* assembly of the ancient genome itself and the negative control was an *Escherichia coli* O157 plasmid. A full list of gene names is included in Table S4.

We undertook visual investigation of the capsular locus using Artemis v18.1.0 [36]. We aligned the ancient metagenome to several representatives of extant diversity using a custom mapping, variant calling and local realignment around indels pipeline using bwa-MEM v0.7.17 [14], and samtools mpileup v1.6 [14]. Reference genomes included the aforementioned PMEN1, TIGR4, Swiss non-typable genomes, and *S. mitis* B6. The boundaries of the capsular locus were defined by the flanking genes *dexB* and *aliA*.

Genes within the capsular locus are 65 % AT-rich, compared to the 60 % of the rest of the genome, resulting in lower sequencing potential. We compared the coverage of the *cps* region to rolling 300 bp regions across the genome with AT content similarity to within 0.5 % of the capsular locus. Coverage per AT content within the *cps* compared to the rest of the genome was calculated using a parametric unpaired *t*-test. We determined the relative risk of low coverage as a result of AT content bootstrapping 200 times to capture 95 % confidence intervals:

(1) $$\frac{CPS_{Depth\;of\;Coverage}/CPS_{AT\;Content}}{Other\;Genome_{Depth\;of\;Coverage}/Other_{AT\;Content}}$$

Risk of given depth of coverage with respect to the AT content in each region. *cps*: capsular polysaccharide locus, Other: The entire genome excluding the capsular polysaccharide locus. Depth of Coverage was calculated by bedtools v2.29.

## Results

### Ancient metagenome

In brief, the initial ancient metagenomic data were received from the University of Copenhagen and as described in Jensen *et al.* [22]. They removed all human reads by aligning to hg19, and we removed any remaining human reads with *H. sapiens* taxonomy IDs. The kraken results included *S. pneumoniae* and *S. mitis* species in the top ten hits (Table S1).

### Pneumococcal metagenome

Extracting the reads which aligned to a curated pneumococcal database of 20.05k pneumococcal genomes yielded 2 186 583 reads with lengths ranging from 19 to 81 nt. The average mapping quality (ratio between the sum of base qualities and total length) was 39.5. The average read length was 55.5 bases and the overall GC content was 41 %.

The megahit assembly was highly fragmented with a longest contiguous sequence of 2362 bp, N50 of 657 and GC content of 38.97 %; the GC content of a typical pneumococcal genome is 39.6 % [23]. The total length of the *de novo* assembly was 340 964.

After filtering the SNPs, as described in the Methods, and aligning to pneumococcal serotype 23F (PMEN1) there were 2729 heterozygous sites. The heterozygous SNP site to homozygous SNP site ratio (het/hom) was 0.9. In the global pneumococcal sequencing project, the het/hom cutoff used for contamination is 0.015. A 0.9 het/hom ratio indicates a mixed sample [23, 27].

### Streptococcal metagenome

The high rates of within- and between-species recombination in Mitis group streptococci make the likelihood of disentangling the genes of a specific species dubious. Therefore, we worked to acquire all genes present in this ancient metagenome which might belong to any streptococcal Mitis group species. We did this by including Mitis group species in our previously pneumococcal exclusive database to extract a *Streptococcus* metagenome. This yielded 2 481 800 reads with lengths ranging from 19 to 81 bases. The average read length was 55.6 bases and the GC content was 41 %.

The megahit assembly was again highly fragmented. The largest contig was 2462 bp, the N50 was 660 and the GC content was 38.74 %. The total length of the *de novo* assembly was 354 960.

**Fig. 2.**
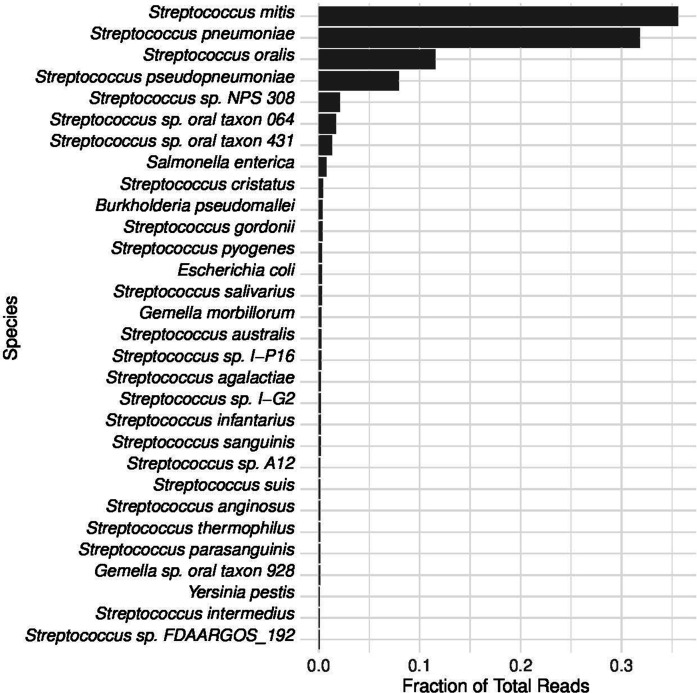
Genomic reads extracted from alignment to a streptococcal database binned by kraken2 v2.0.8 and abundance estimated by bracken v2.5.2. In total, >96 % abundance is attributed to Mitis group streptococcal species.

There were 2707 heterozygous SNP sites when we aligned the ancient metagenome against pneumococcal serotype 23F (PMEN1) with a het/hom ratio of 0.9, and there were 3248 heterozygous SNP sites when we aligned it against *S. mitis* B6 with a het/hom ratio of 1.1. These ratios are highly indicative of a mixed sample. In total, 96.2 % of the reads were classed as belonging to the genus *Streptococcus* (Fig. 2).

Extraction using the streptococcal database resulted in 96.38 % of reads belonging to *Streptococcacae*. Of these 0.15 % were identified as streptococcal phages.

The mapDamage reports for both ancient metagenomes indicated ancient DNA damage including 5′ C to T and 3′ A to G substitutions (Fig. S1) and accumulation of transitions according to read length.

### Contextualization within extant streptococcal phylogenies

**Fig. 3.**
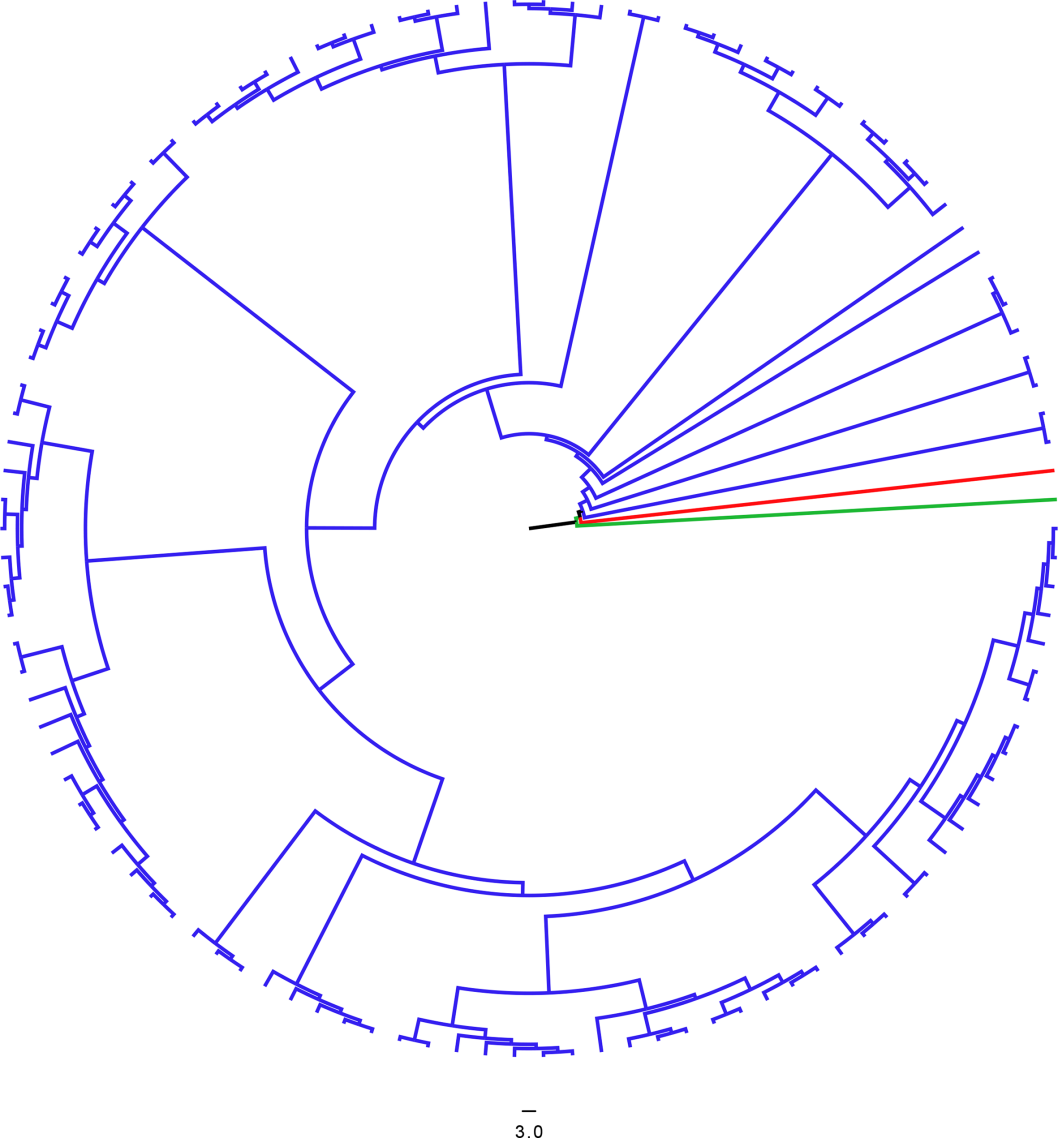
Ancient metagenome (red) in the context of the diversity of dominant (GPSCs with over 100 identified strains) extant pneumococcal strains (blue) rooted to an *S. mitis* outgroup (green). The tree was built using IQ-TREE with 1000 bootstraps and a GTR model. Scale bar represents nucleotide substitutions per site.

We included the ancient metagenome extracted from the exclusively pneumococcal database in a phylogenetic tree with a representative of each extant pneumococcal GPSC (*N*=785), and an *S. mitis* outgroup. The resulting multi-fasta alignment length was 2 221 315 bp with 409 919 SNP sites when including *S. mitis,* and 76 685 SNP sites when excluding S. mitis*.* The mean SNP distance between the ancient genome and pneumococcal strains was 7 444 61 bp. The ancient metagenome reads covered 88.23 % of the reference genome. The ancient metagenome does not lie within extant pneumococcal diversity (Fig. 3).

**Fig. 4.**
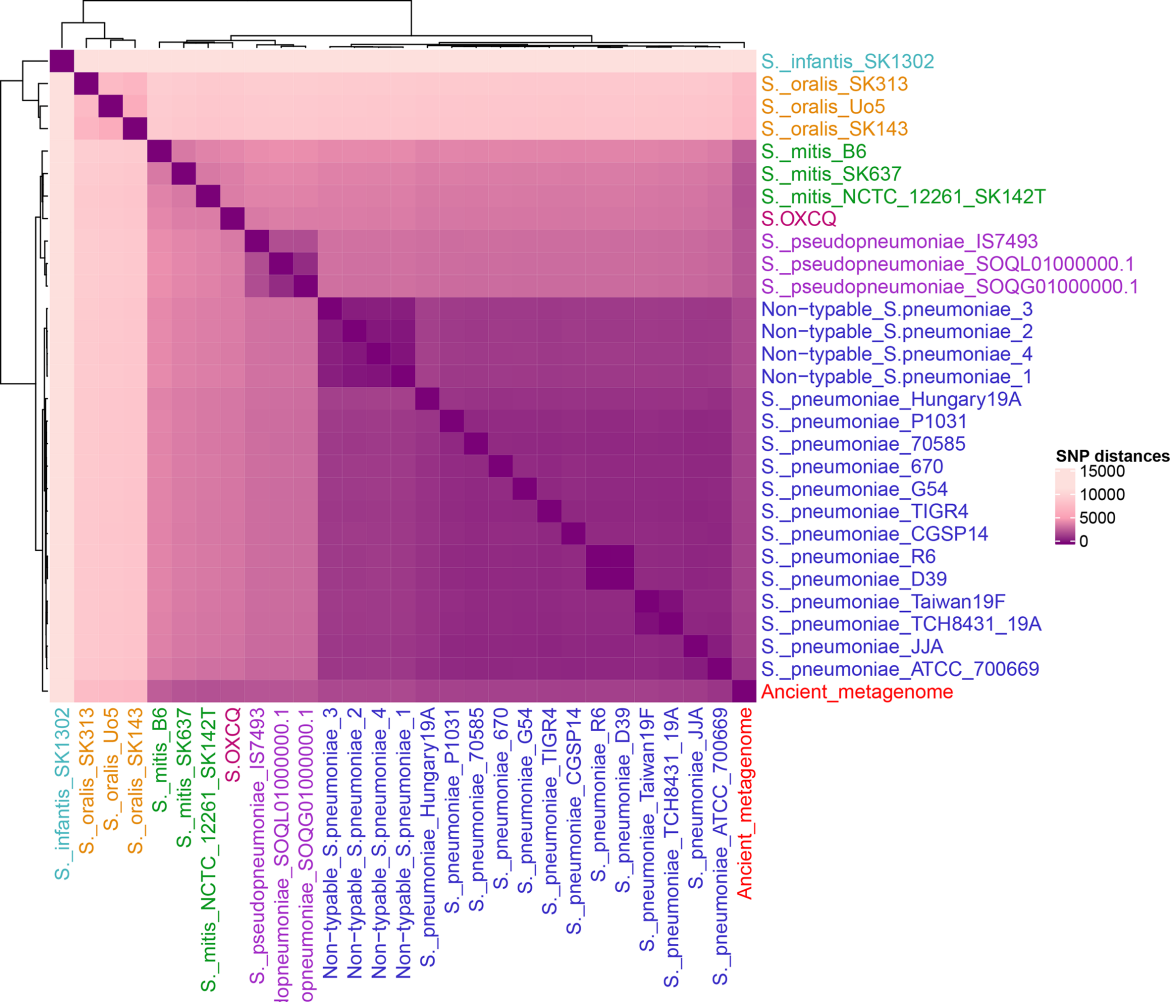
SNP distance heatmap of representative genomes from Mitis group species including *S. infantis, S. oralis, S. mitis. S.* OXCQ*, S. pseuodpneumoniae*, four non-typable *S. pneumoniae* represenatives, and *S. pneumoniae *. Colouring is as follows: *S. pneumoniae* (blue), *S. pseudopneumoniae* (purple), *S. mitis* (green), *S. infantis* (turquoise), *S. oralis* (orange), *S. OXCQ* (pink), and the Ancient metagenome (red). *S.* OXCQ is a novel, pathogenic, Mitis group species in the streptococcal database described by Kirkeleite *et al*. [24]

**Fig. 5.**
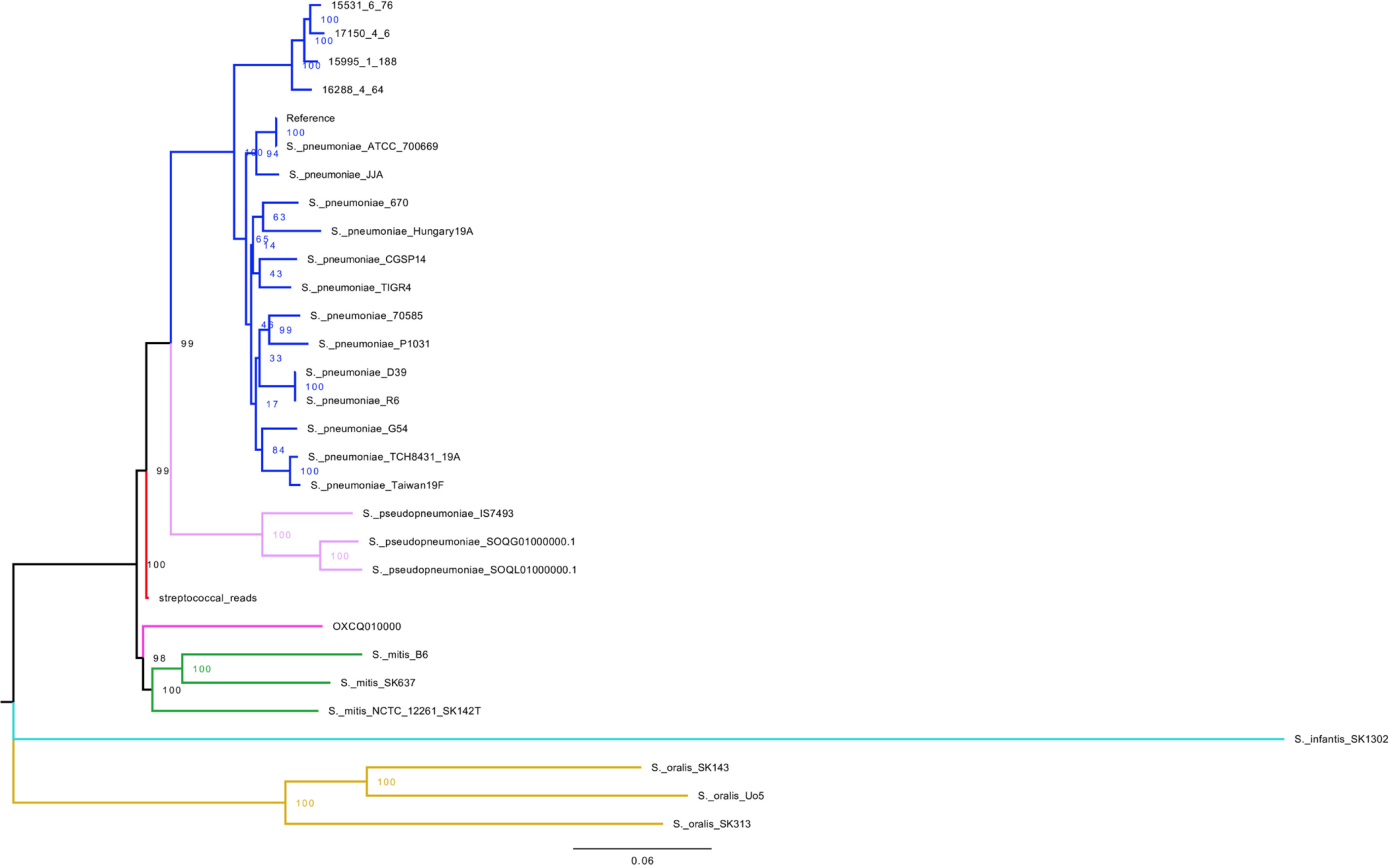
Ancient metagenome within the diversity of extant Mitis group species with IQ-TREE bootstrapped confidence values. The tree was built with IQ-TREE with 1000 bootstraps and a GTR model. The tree is rooted to the most distantly related of these, *S. infantis *, and visualized using FigTree. Scale bar represents nucleotide substitutions per site. Colouring is as follows: *S. pneumoniae* (blue), *S. pseudopneumoniae* (purple), *S. mitis* (green), *S. infantis* (turquoise), *S. oralis* (orange), *S.* OXCQ (pink), and the Ancient metagenome (labelled streptococcal reads) (red). The top four *S. pneumoniae* species are non-typable (contain no capsule). *S.* OXCQ is a novel, pathogenic, Mitis group species in the streptococcal database described by Kirkeleite *et al* [24]. All accession numbers are described in Table S9.

Due to the presence of multiple streptococcal species and their abundance in the metagenome, we wanted to determine if the ancient streptococcal metagenome sits within the diversity of any extant streptococcal species. We aligned the reads extracted from the streptococcal database to Mitis group species (*mitis, oralis, infantis*), *S. pseudopneumoniae *, *S.* OXCQ and 18 extant pneumococcal genomes, using PMEN1 as a reference genome. This resulted in a multi-fasta alignment length of 2 221 315 and 28 370 variable sites. The mean SNP distances between the ancient genome and other species were 1558.5 for pneumococcus, 1911.8 for non-typable pneumococcus, 2360.7 for *pseudopneumoniae*, 2275 for *S.* OXCQ, 2392.7 for *mitis*, 7446.7 for *oralis*, and 11 255 for *infantis* (Fig. 4). The ancient metagenome does not sit within the diversity of any specific extant streptococcal species, or any other known species. Its smallest SNP distance is to *S. pneumoniae* and it lies between the *S. pseudopneumoniae* and *S. mitis* groups in the IQ-TREE phylogeny. Our 1000 bootstraps conferred 100 % confidence for the ancient metagenome node (Figs 5 and S2).

The pseudo-mixed, extant, *S. mitis* and *S. pneumoniae* reads fit within the diversity of *S. pneumoniae.* This implies that if *S. pneumoniae* were present it would likely be within the extant species diversity when aligned to an *S. pneumoniae* reference genome (Fig. S3).

When all transitions were removed, metagenomes from both the pneumococcal database (Fig. S4a) and the streptococcal database (Fig. S4b) have common ancestors with *S. mitis, S. pseudopneumoniae* and *S. pneumoniae.*

It is likely that multiple streptococcal species or multiple strains of the same species are included in this metagenome. Regardless, as expected of a highly recombinant genus such as *Streptococcus *, this 5700-year-old metagenome does not fit within any known extant species. Furthermore, due to the high rates of recombination within and between species it is impossible to disentangle exactly which Mitis group species combinations may have been present in the oral cavity of this ancient human [22].

### Absence of genes for polysaccharide capsule biosynthesis in the ancient streptococcal metagenome

Our work to identify a capsule amongst the ancient metagenome reads identified no capsular types with minhash scores greater than 3/1000 for similarity to the ancient metagenome. Furthermore, SRST2 interrogation of 95 serotypes identified the closest capsular type to be *aliB,* which is present only in non-encapsulated pneumococci and other Mitis group species [35].

**Fig. 6.**
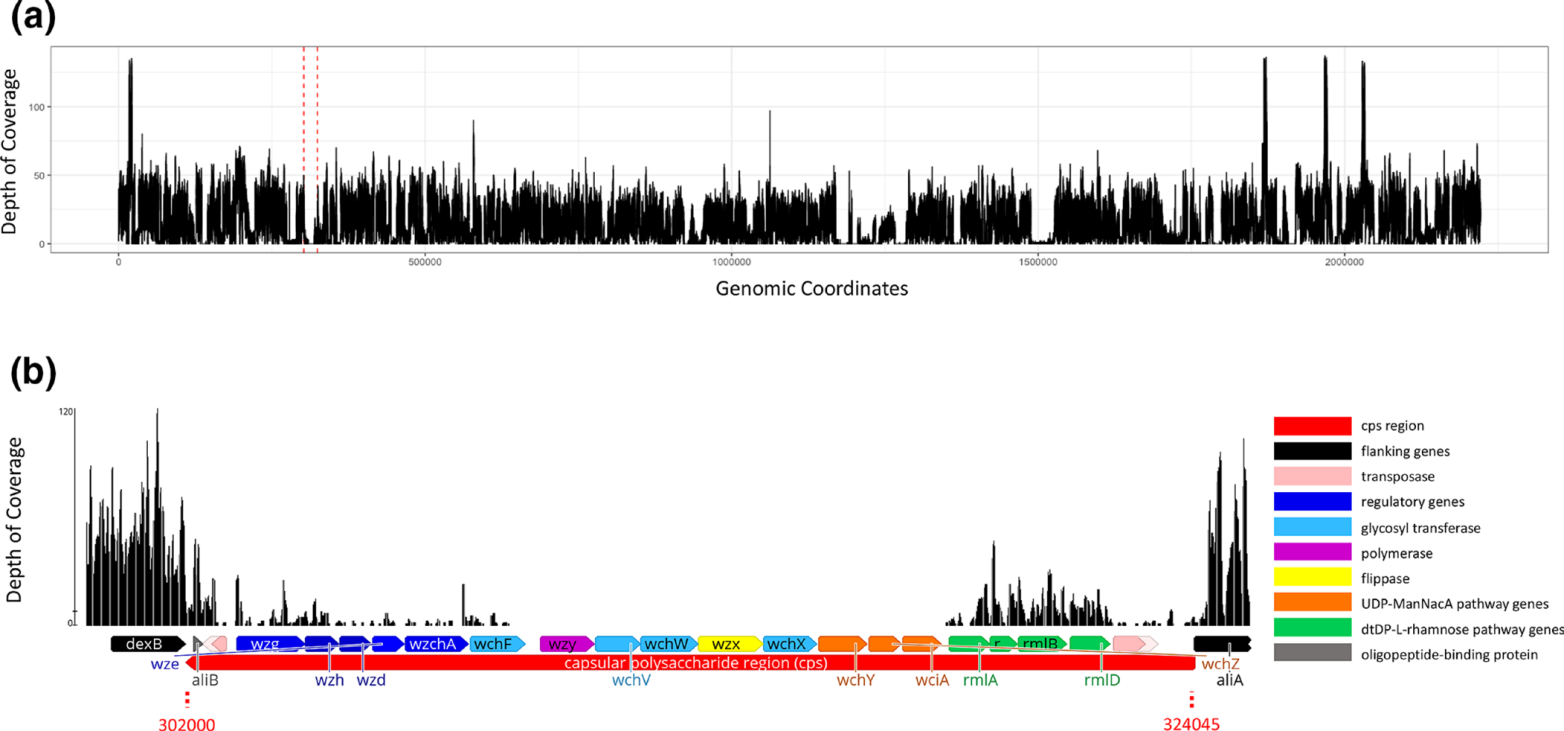
Depth of coverage of the ancient metagenome. (a) Depth of coverage of the entire genome aligned to PMEN1. Vertical red dashed lines indicate the location of the capsular polysaccharide region. (**b**) Ancient metagenome depth of coverage alignment to PMEN1. The capsular polysaccharide locus is noted in red, and the genomic coordinates of the cps region are marked with red dashed lines at 302008–324045. Genes are coloured according to function and include flanking genes (black), transposases (light pink), regulatory genes (dark blue), glycosyl transferases (light blue), polymerase (purple), flippase (yellow), UDP-ManNacA pathway genes (orange), dTDP-l-rhamnose pathway genes (green) and oligopeptide binding protein aliB (grey). The figure was visualized in Geneious v2021.2.2. The colour scheme is adapted from Bentley *et al*. [34].

Aligning the ancient metagenome against the *cps* gene database demonstrated the presence of the flanking genes, *dexB* and *aliA*, and low-coverage mapping, minimum read depth of 10, against genes indirectly associated with capsular polysaccharide biosynthesis: *wzh*, *rmLB*, *rmlC* and *wzd* (Table S3). Additionally, transposases typically found within the *cps* locus of extant genomes IS*1167* and IS*630* were present, although they have no known involvement in capsule biosynthesis (Table 1) [37–39] (Fig. 6). The coverage statistics for the capsular genes with streptococcal reads mapping to them and the full contents of the capsular gene database are available in Table S4 [24].

**Table 1.** ARIBA *cps* locus gene hits Columns include gene name, reference genome, number of reads mapping to the gene, reference length, percentage identity, contig length and average depth of coverage

Gene name	Reference	Ref. contig length	Reads mapping	Identity (%)	Contig length (bp)	Average coverage
dexB*	PMEN1	1608	510	96.63	386	18.03
aliB*	PMEN1	174	1728	94.48	330	17.50
*rmlA*	PMEN1	870	14	95.68	140	2.30
*rmlC*	PMEN1	594	48	98.37	144	9.60
*rmlB*	PMEN1	1050	124	98.08	130	11.53
*rmlD*	PMEN1	852	72	98.36	428	9.00
aliA*	PMEN1	1983	484	96.45	479	32.06
IS*630-Spn1*, transposase Orf2*	TIGR4	339	124	95.27	328	21.30
IS*630-Spn1*, transposase Orf2*	TIGR4	348	80	97.33	150	12.70
*cps4D*	TIGR4	684	28	97.47	158	5.30
IS*1167*, transposase, truncation	TIGR4	102	182	96.3	87	59.80
Positive control*	*De novo* assembly ancient	1386	830	99.64	1406	35.30
Positive control*	*De novo* assembly ancient	509	140	100	503	17.00
Positive control*	*De novo* assembly ancient	688	588	98.95	668	51.30
Positive control*	*De novo* assembly ancient	1583	580	99.6	995	22.97

*Those with an average depth of coverage >10.

Fewer reads map to the *cps* region than is expected when compared to similarly AT-rich regions across the rest of the ancient genome. Using a parametric unpaired *t*-test, the coverage of the capsular region is less than can be explained by the AT content alone (*P*=2.2E-16), indicating that the absence of mapping is not an artefact caused by failure to map to AT-rich sequences. The relative risk of this low depth of coverage explained by AT content is 0.16 [95 % confidence interval (CI) 0.03–0.37] (Fig. S5).

Visual investigation of the capsular locus alignments revealed the low coverage elucidated previously by the capsular database. The ancient reads mapped to some of the genes typically found in the 5′ end of the *cps* locus and some mapped to the *rml* genes (typically 3′). Notably, the genes central to polysaccharide biosynthesis (glycosyl transferases, flippase and polymerase) had no reads mapping to them (Fig. 6).

The presence of *cps* flanking genes, absence of mapping to *cps* genes and inability to explain the absence by AT-rich sequencing error together imply absence of the *cps* locus within this ancient metagenome.

### Presence of virulence genes within the ancient streptococcal metagenome

The ancient metagenome contained 57 genes which Kilian and Tettelin [30] previously identified as being exclusive to *S. pneumoniae,* using a threshold of 20 % for the fraction of sites per read with non-zero coverage. These included pneumolysins, bacteriocins and hyaluronidases (Table S5). Note that this previous analysis was performed with modest numbers of genomes and thus some of these genes could be present, if only rarely, in other species.

Coverage of the ancient metagenome against TIGR4 and PMEN1 zinc metalloprotease (*zmp*) genes demonstrated the presence of *zmpA* with a mean mapping coverage of 86.78 %, *zmpD* with a mean mapping coverage of 71.54 % and *zmpC* with a mean mapping coverage of 37.35 %. The mean mapping coverage for *zmpB* was 3.36 % and the mapping coverage against *zmpB* did not exceed 12 % in any instance (Table S6). This implies that it is unlikely *zmpB* is present amongst these ancient metagenome reads and may counter the previous assertion that *zmpB* is the ‘ancestral *zmp*’ as was postulated by Bek Thomson *et al* [40].

Several virulence factors and antimicrobial resistance genes were detected by mapping to CARD and VFDB. These included the 26 pneumococcal virulence factors identified by Jensen *et al.* [22] such as IgA1 protease, streptococcal enolase, neuraminidase and multiple choline binding proteins (Fig. S6). Although there are hits to metabolic genes typically associated with the capsule, these genes are not sufficient for capsular synthesis. Furthermore, similar metabolic genes are present elsewhere in the genome, allowing for the possibility that these are mis-mapping (Tables S7 and S8).

## Discussion

Here we confirm previous findings by Jensen *et al.* [22] that approximately 5700 years ago the respiratory tract of a woman in Syltholm on the island of Lolland in Denmark was probably colonized by some streptococcal bacterial species. However, although phylogenetically these genome reads sit within the diversity of the Mitis group streptococci they do not sit specifically within the diversity of any of the extant Mitis group species (* S. pneumoniae *, *S. pseudopneumoniae *, *S. mitis *, *S. oralis *, *S. infantis *), or novel *S.* OXCQ. The heterozygosity and high rates of recombination known to streptococci means that the metagenome may include multiple taxa. The phylogenetic position of the ancient metagenome shows no bias towards any extant species and probably includes a common ancestor to *S. pneumoniae* and *S. pseudopneumoniae *, or a common ancestor between these and *S. mitis.* Furthermore, the position in the phylogeny of the extant species *S.* OXCQ suggests that it may be a persisting close relative of the ancient taxon/taxa. It can be expected that the increasing availability of aDNA will improve our ability to resolve the phylogenetic positions of such taxa [25, 26].

There are four known streptococcal zinc metalloprotease (*zmp*) genes, *zmpA* (commonly referred to as iga), *zmpB*, *zmpC* and *zmpD.* The genes *zmpA* and *zmpB* are known to cleave the primary mediator of human mucosal immunity, IgA1, and are present in the three primary bacterial species causing of meningitis: *Neisseria meningitidis, S. pneumoniae* and *Haemophilus influenzae* [20, 27, 28]. It has been postulated that these proteases were integral to the diversification of *S. pneumoniae* and *S. mitis* [7], in which the pneumococcal *zmp* genes diversified, increasing the ability to evade the adaptive immune system. ZmpC cleaves human matrix metalloproteinase 9 (MMP-9) while the substrate and function of ZmpD are undefined [29, 30]. Bek Thomson *et al.* [40] found that *zmpA* and *zmpB* were present in all 67 examined pneumococcal strains with *zmpB* present in all examined *Streptococcus* except for *Streptococcus suis; zmpC* is variably present across investigated *Streptococcus* while *zmpD* is only present in the pneumococcus and *S. mitis *. The read coverage of the ancient metagenome at these gene regions is variable, with >20 % coverage in *zmpA*, *zmpC* and *zmpD*, and absence in *zmpB* (Table S6).

The presence of *zmpA* in the ancient metagenome indicates adaptation to human mucosal immunity, confirming its probable adaptation to an ancient human niche. The presence of *zmpD* is concordant with the assertion that this ancient metagenome includes a common ancestor to *S. mitis* and the pneumococcus, or the pneumococcus and *S. pseudopneumoniae *. The absence of *zmpB* draws into question the hypothesis that *zmpB* is the ancestral *zmp* [20]. If these ancient reads are representative of *Streptococcus* at that time, *zmpB* may have been imported to the pneumococcus and *S. pseudopneumoniae* more recently than 5700 years ago.

The capsule biosynthesis locus is prevalent among extant streptococcal species, though rarer non-encapsulated strains coexist. The capsule is thought to facilitate nasopharyngeal carriage with capsule size correlated with duration of carriage [31]. Our knowledge of the existence of NESp has gained prominence due to implementation of vaccines targeting the capsule antigen, and as a result of the development of more sensitive detection techniques, including genome sequencing [5, 32, 33]. Two groups of NESp have been characterized: group I has a non-functional *cps* locus (normally due to complete or partial deletion), while for group II the *cps* locus is replaced by a variety of combinations of *pspK* and *aliB*-like genes, typically two *aliB*-like ORFs [34, 35]. Although *aliB*-like genes are entirely absent from encapsulated pneumococcal genomes, they are present in other species of encapsulated streptococci [2]. The group II NESp have been further classed into capsule-null clades 1, 2a, 2b and 3, depending on the combination of *pspK* and *aliB*-like genes present [5]. The absence of a capsule reduces the average carriage duration time of NESp and is proposed to be compensated for by increased presence of other surface proteins including pneumococcal surface proteins and pneumolysins. NESp have been hypothesized to inhabit a niche distinct from that of encapsulated pneumococci [34]. Furthermore, although a typical *cps* locus has been observed in *S. pseudopneumoniae *, many have a locus similar to a specific type of non-encapsulated pneumococcal strains (NCC3), which includes *dexB*, *aliB* genes and a *glf*; these also lack the downstream *aliA* [36].

The ancient reads include the conserved *dexB* and *aliA* flanking regions but have no mapping to any of the polysaccharide biosynthesis genes normally contained in the *cps* locus of streptococcal species. Furthermore, we did not identify any of the alternative loci observed in extant NESp genomes. If the ancient metagenome is representative of the *Streptococcus* of the time, and includes an ancestor to extant *S. pneumoniae* and *S. pseudopneumoniae *, this implies either that the *cps* locus was imported to the capsulated strains of these species more recently than 5700 years ago, or that this ancient metagenome only represents non-encapsulated species among a population containing both. The lack of capsule implies that these ancient streptococcal species had relatively short carriage duration and the ancient non-encapsulated Mitis group were probably highly recombinogenic and naturally competent, which is concordant with some reads mapping to competence gene *comC* [33,34]. This also suggests that ancient streptococci were more likely to be commensal species as absence of a capsule implies reduced virulence and pathogenicity [17, 41]. This may have implications for the long-term sustainability for capsule-targeting vaccination. Furthermore, the human population is estimated to have undergone exponential grown from approximately 5000 to 400 BCE (this metagenome is dated to 3679 cal. BCE) [22, 42]. The ancient human expansion may have allowed the ancient *Streptococcus* to encounter more human pathogens and acquire genes, such as *cps*, which are conducive to pathogenicity.

## Conclusion

Our ancient streptococcal metagenome analysis indicates the existence of ancestral species 5700 years ago that are likely to be extinct, but nevertheless related to extant Mitis group *Streptococcus *. The derived metagenome lacks evidence of *cps* genes, suggesting that the *cps* locus may have been imported into the Mitis group more recently than 5700 years ago or that the metagenome sampling does not capture *cps*+ streptococci that were present at that time. The presence of multiple zinc metalloproteases suggests that adaptation to host IgA1 had begun and the presence of other potential virulence factors further implies development of close host–microbe interactions, though the absence of a capsule suggests an inability to cause invasive disease. The presence of specific virulence factors such as pneumolysin implies stable maintenance of such genes through streptococcal evolution that may strengthen their value as anti-pneumococcal vaccine antigens.

Short read length and low read depth made it difficult to confidently conduct complete gene presence/absence analysis, or to disentangle which reads belong to individual species and in turn how many Mitis group ancestors are present in the ancient sample, and where they fit within the diversity. As more ancient metagenomic DNA becomes available, and genome sequencing of aDNA is further improved, it may be possible to confirm or dismiss our conclusions regarding the evolutionary history of these important human pathogens. This may include more targeted interrogation of specific loci and assembly of heterozygous fragmented reads. Improved understanding of historical gene presence among Mitis group streptococci can improve our efforts to mitigate disease caused by these highly recombinogenic dynamic bacterial pathogens.

## Supplementary Data

Supplementary material 1

Supplementary material 2
