# Supplementary material for: A new perspective on ancient Mitis group streptococcal genetics
Source: Microb Genom. 2022 Feb 28;8(2):000753. doi: 10.1099/mgen.0.000753 (PMC8942026; doi:10.1099/mgen.0.000753)
Supplement: Supplementary material 1 [file mgen-8-0753-s001.pdf]

Supplementary Figures

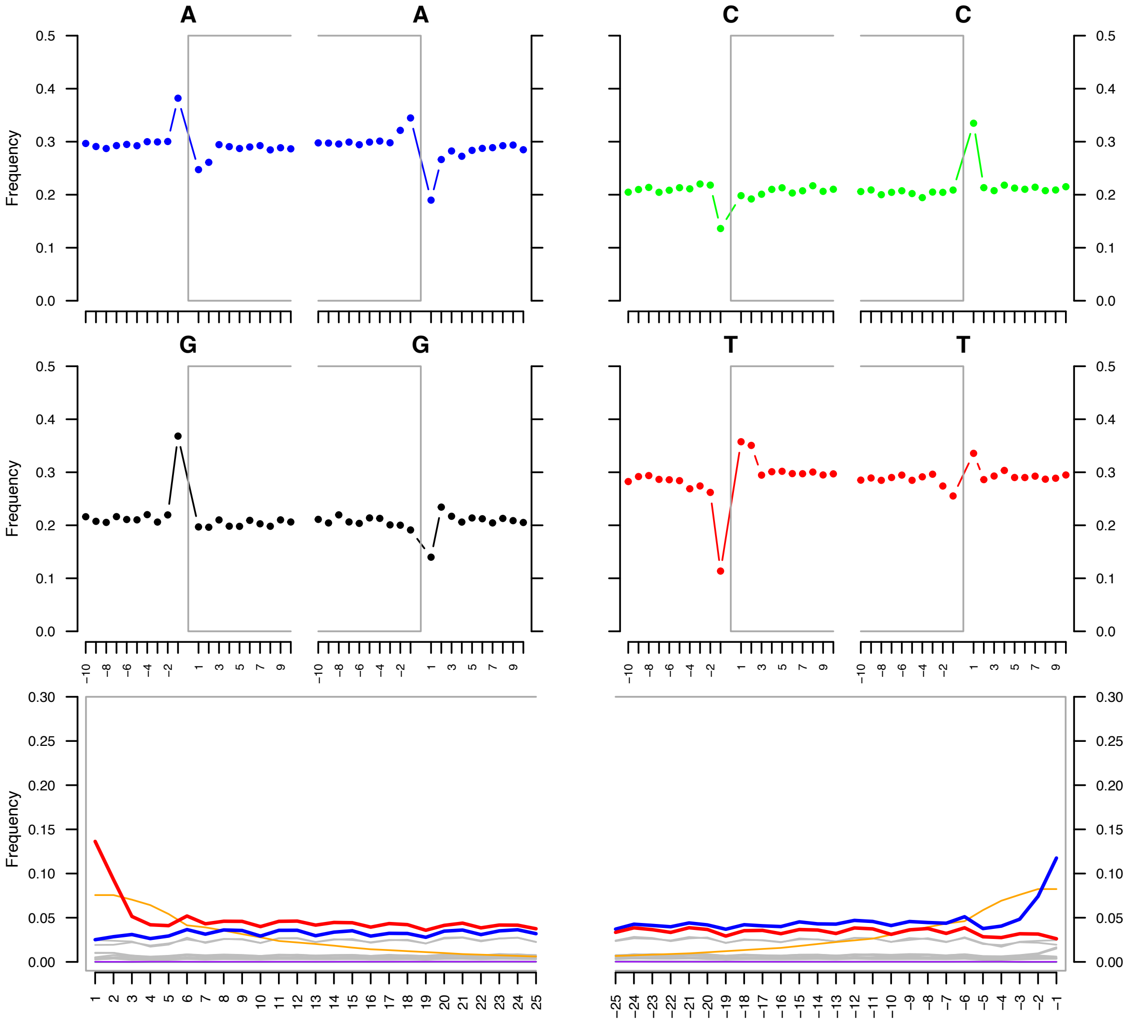

Supplementary Figure 1. mapDamage misincorporation report characteristic of ancient DNA. The four upper mini-plots show the base frequency outside and in the read (the open grey box corresponds to the read). The bottom plots are the positions' specific substitutions from the 5" (left) and the 3" end (right). The following color codes are used in the bottom plots: C to T substitutions (Red), G to A substitutions (Blue), All other substitutions (Grey), Soft-clipped Bases (Orange), Deletions relative to the reference (Green), Insertions relative to the reference (Purple).

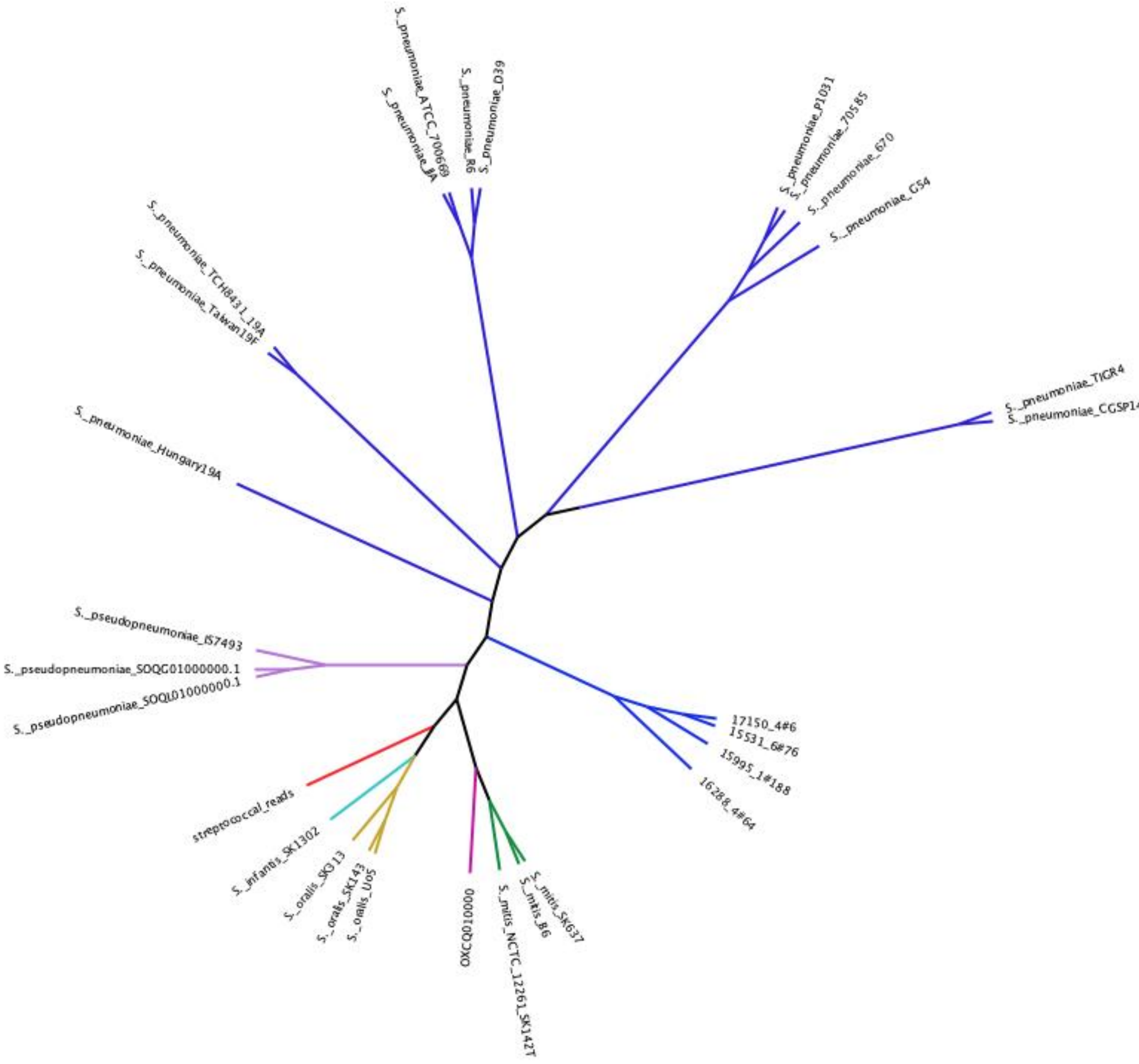

Supplementary Figure 2. Contextualization of 5700 year-old genome among extant pneumococcal and Mitis group species. In an unrooted phylogenetic tree including Streptococcus pneumoniae (blue), Streptococcus pseudopneumoniae (purple), Streptococcus mitis(green), Streptococcus infantis (turquoise), Streptococcus oralis (mustard yellow), S. OXCQ (dark purple/pink), and the ancient metagenome (red). This tree was using IQtree with 1000 bootstraps and a GTR model. Scale bar represents nucleotide substitutions per site.

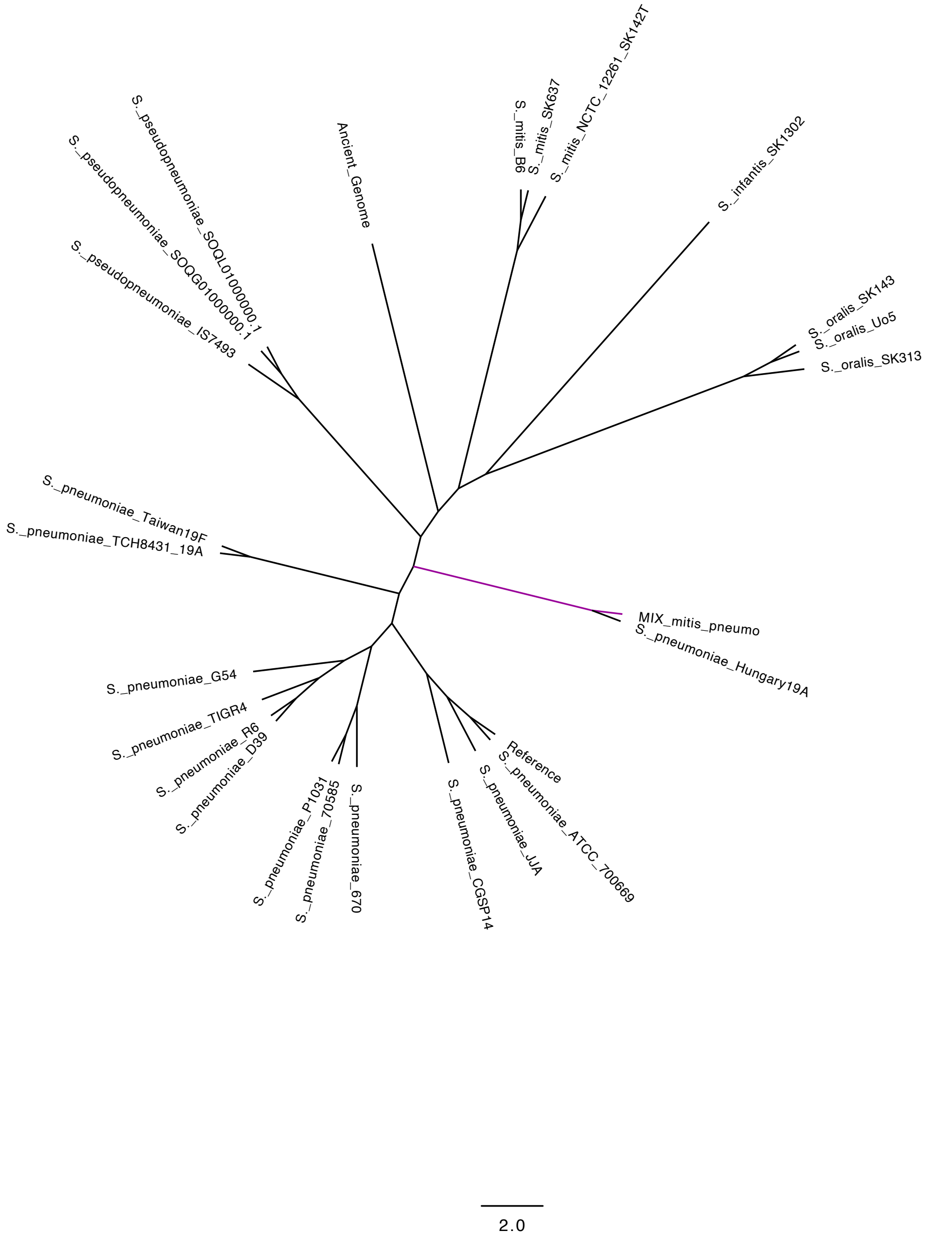

Supplementary Figure 3: Inclusion of a pseudo-mixed mitis and pneumococcal genome (purple) called "Mix_mitis_pneumo" included in the previous context tree described in Supplementary Figure 2. This tree was built with iqtree with 1000 bootstraps and a GTR model. Visualized as an unrooted tree. Scale bar represents nucleotide substitutions per site.

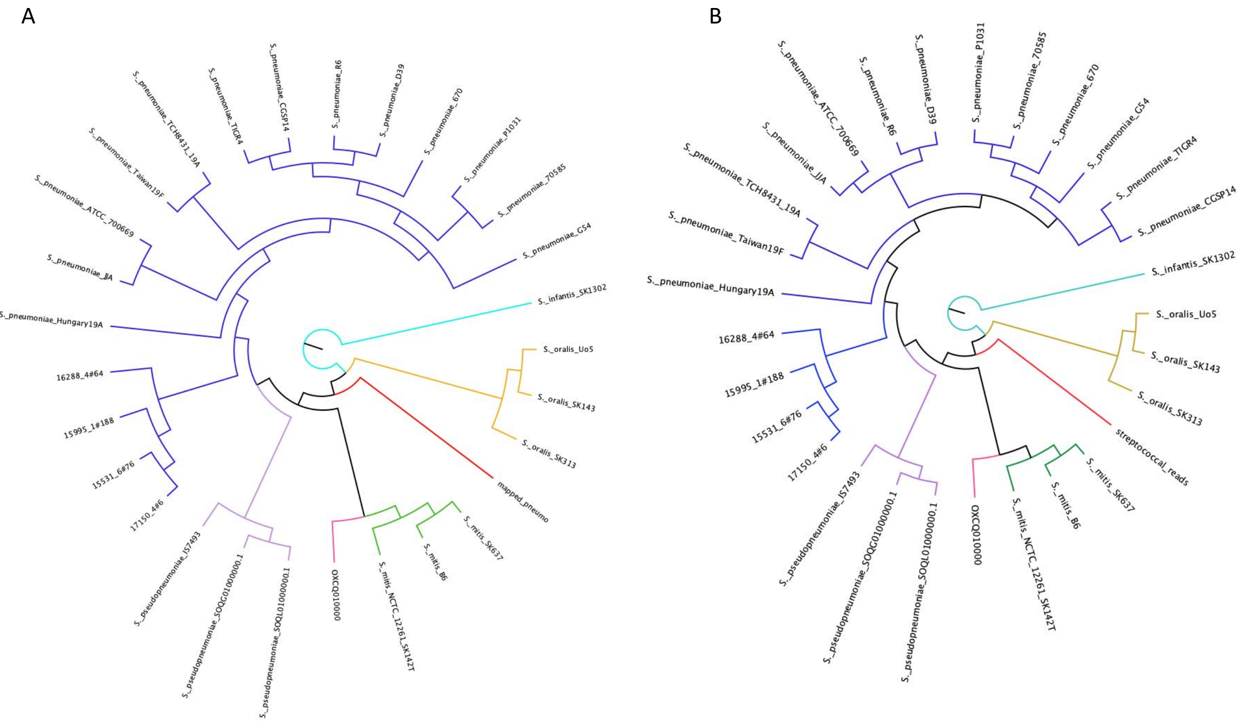

Supplementary Figure 4. Ancient streptococcal metagenome within the diversity of extant Mitis group species without transitions. Tree built with vcf-kit with all transition substitutions removed from the multi-fasta SNP alignment. The tree is rooted to the most distantly related of these, Streptococcus infantis and visualized using FigTree. A) includes ancient metagenome extracted from alignment to the pneumococcal database. B) includes ancient metagenome extracted from alignment to the streptococcal database. Coloring for both A and B is as follows: Streptococcus pneumoniae(blue), Streptococcus pseudopneumoniae(purple), Streptococcus mitis(green), Streptococcus infantis (turquoise), Streptococcus oralis(orange), S. OXCQ (pink), and the Ancient metagenome (red).

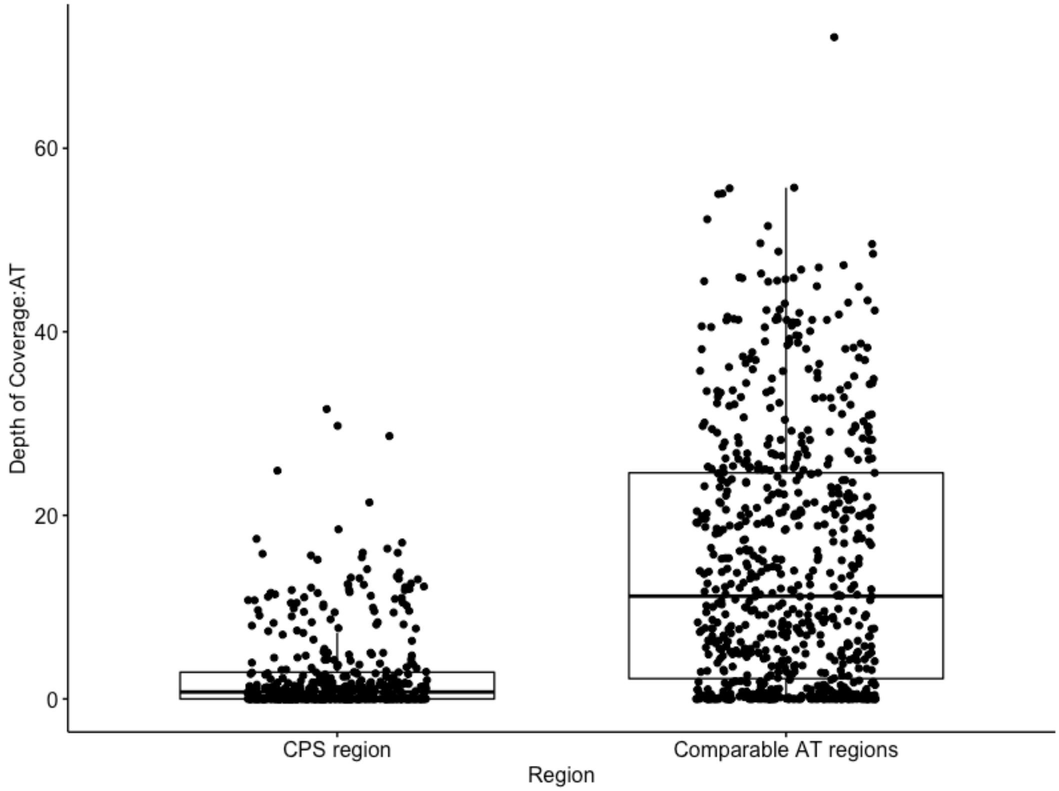

Supplementary Figure 5. Depth of Coverage per AT% cps region of the Ancient metagenome Boxplot of Depth of Coverage per Average AT of rolling 300bp scaffolds within the CPS region (left) and across AT regions within 0.5% of the CPS regions AT content across the rest of the genome (right). p-value 2.2E-16

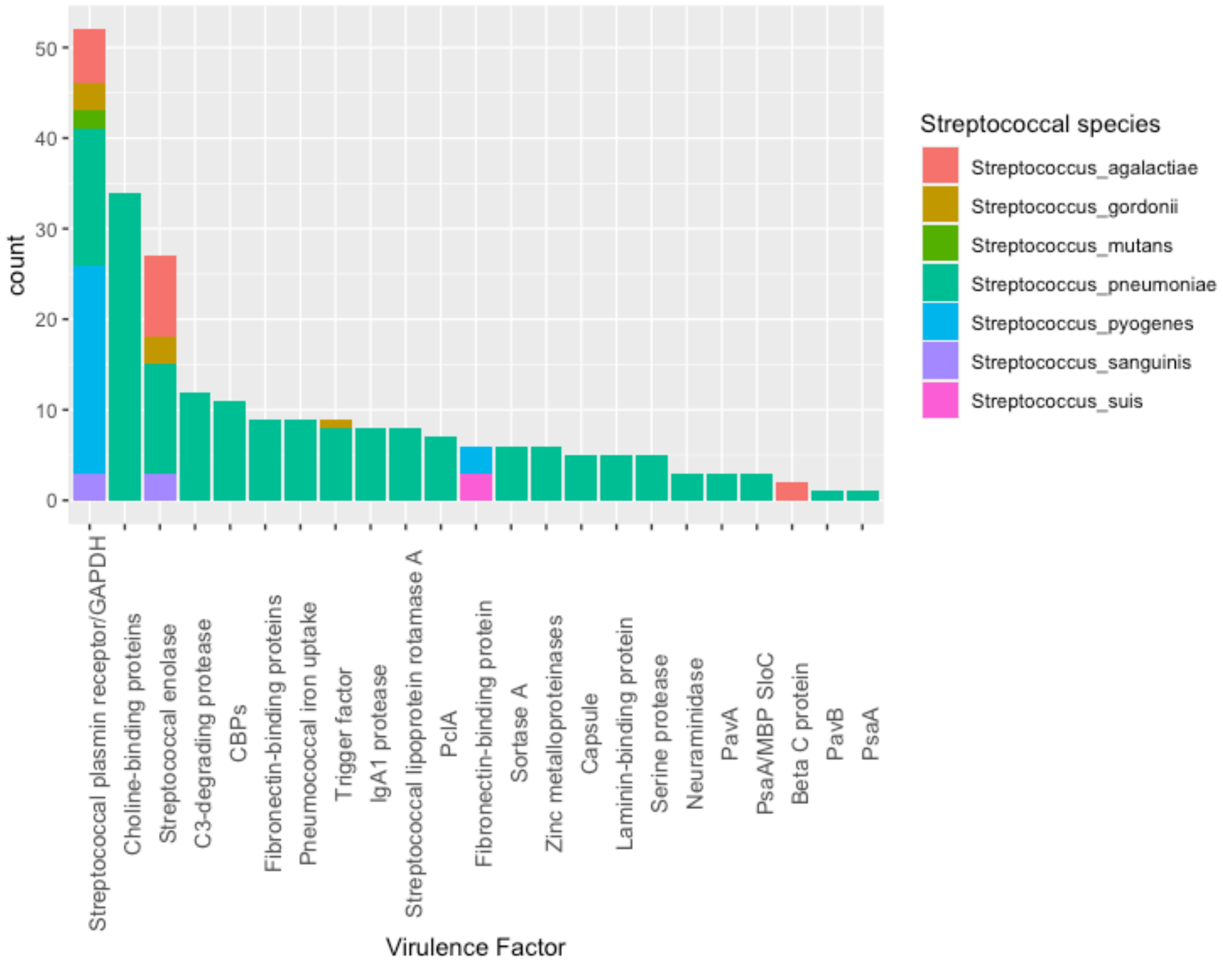

Supplementary Figure 6. Virulence Factor Database hits. Colored by streptococcal species with the highest match to the ancient metagenome. These were filtered by bitscore > 200, coverage >20%, and identity of >80%.

Supplementary Tables

Supplementary Table 1. Raw output from kraken and bracken classifying the species to which the ancient metagenome reads have the highest identity after removal of human reads. Ordered descending by fraction of total reads assigned to each species

Supplementary Table 2. Raw output from kraken and bracken classifying the species to which the ancient metagenome reads have the highest identity after extracting from the streptococcal database. Ordered descending by fraction of total reads assigned to each species

Supplementary Table 3. Raw output from ARIBA. Column labels are described in ARIBA wiki manual

Supplementary Table 4. Reference genes included in the capsular gene database interrogated using ARIBA.

Supplementary Table 5. Prevalence of streptococcal genes for S. pneumoniae, S. pseudopneumoniae, S. mitis, S. oralis, and the ancient metagenome extracted from the pneumococcal database and the streptococcal database. Coverage calculated using bedtools v2.29. Species prevalence determined in Kilian & Tettelin et.al. (2019)

Supplementary Table 6. Coverage of ancient metagenome against zmp genes using bedtools v2.29.

Supplementary Table 7. Output from the Comprehensive antibiotic resistance database (CARD). All were loose hits with at least 95% identity using a strict cutoff.

Supplementary Table 8. Virulence Factor Database (VFDB) raw output from BLAST search using ancient metagenome reads.

Supplementary Table 9. Streptococcal reference genome metadata.
